# Investigating Hip Arthroplasty Femur Preparation Training Using a Haptic-Enabled Virtual Reality Simulation

**DOI:** 10.1177/15533506251383830

**Published:** 2025-10-03

**Authors:** Justin M.T. Duncombe, Pierre Camaly de Brosses, Al-Amin M. Kassam, David J. Harris, Gavin Buckingham

**Affiliations:** 1 Department of Public Health and Sport Sciences, University of Exeter Medical School, 3286 University of Exeter, Exeter, UK; 2 Learning Experiences and Innovation, 3286 University of Exeter, Exeter, UK; 3 Exeter Hip Unit, Royal Devon University Hospital, Exeter, UK

**Keywords:** hemiarthroplasty, medical education, immersive technology, VR, surgical skills

## Abstract

**Objective:**

Virtual reality (VR) simulation training offers a promising solution to the growing challenges of acquiring operative experience in surgical skill development. As previous studies have primarily utilised VR systems without haptic feedback, there remains limited evidence on the impact of more immersive, tactilely responsive platforms. This study aimed to assess if haptic-enabled VR technology could accelerate the acquisition of hip arthroplasty skills.

**Methods:**

Twenty undergraduate medical students (12 Female, 8 Male; age = 20 ± 2 years) were randomly allocated to either a 60-minute haptic VR training session or a traditional mentor teaching session on hip arthroplasty. After training, all participants performed a SawBone simulated hemiarthroplasty procedure in a physical environment. Outcomes measured included implant depth error, which determined procedural success, operative time, and an objective evaluation of technical skills by a blinded Consultant Orthopaedic Surgeon.

**Results:**

We observed no difference in levels of implant depth error (*P* = .705), rated technical skill (*P* = .704), or operative time (for successful implant insertions; *P* = .551) between traditional and VR-trained groups.

**Conclusions:**

These results indicate that VR may, at least, serve as a valuable adjunct to traditional early-stage training in complex open procedures like joint arthroplasty. The study also emphasized the importance of realistic VR training modules and illustrated the potential limitations of incorporating low-fidelity haptic feedback in VR training for such procedures.

## Introduction

Recent changes in postgraduate medical education in the United Kingdom, driven by government-led initiatives, have led to a restructuring of the training process for surgical trainees. This restructuring has resulted in shorter training periods and reduced operative experiences.^1^ Consequently, the proportion of orthopaedic procedures performed by trainees has dropped from 38% to 17% of cases.^2^ These changes can be attributed to the European Working Time Directive, which limited trainee working hours to a maximum of 48 per week, as well as various initiatives and pressure to reduce long waiting lists for surgery, which all puts pressure on hospitals to increase their surgical output. The COVID-19 pandemic has significantly reduced surgical training opportunities, with the majority of trainees having fewer chances to operate as the primary surgeon.^3-5^ Procedures performed by individual junior trainees declined by up to 64% as the surgical workforce adapted to a global shift towards service provision.^4,6^ This resulted in the centralisation of services, the postponement of elective operations, and the restriction of emergency surgical procedures for higher-risk patients to consultants or senior trainees.^4,7,8^ As a result, traditional operating room teaching opportunities have significantly declined; trainees typically take longer to complete surgical procedures compared to consultants, and so hospitals are under pressure to prioritise theatre efficiency over teaching in order to meet these new demands.^2,3,6-9^

Simulations are increasingly being utilised as an alternative to traditional theatre teaching in order to develop surgical skills.^10^ Low-fidelity models, such as SawBones and cadavers, are valuable tools in medical education, enabling trainees to hone their surgical skills without risking patient safety.^11^ Simulations have proven to be so effective that they have been incorporated into international training curricula.^12,13^ However, SawBone simulations do not fully replicate the real operating room environment or the complexities of soft tissue in surgery, while cadaveric dissections are costly, time-consuming, and non-reusable with significant logistic difficulties.^14^ In response, ongoing efforts are focused on creating higher fidelity simulation models incorporating virtual reality (VR) technologies and software that more closely mimics the actual surgical environment.^15^ Subsequently, these advancements are proposed to further improve the efficacy of simulations in surgical education and training, potentially facilitating improved skill acquisition and subsequent improvement in patient outcomes.^16^

Immersive VR refers to a computer-simulated environment that allows real-time interaction with computer-generated information through normal sensorimotor processes.^17,18^ In the field of orthopaedic surgery, VR training has previously been largely focused on joint arthroscopy.^19^ This focus is attributed to the procedural and instrumental complexity involved in creating realistic simulation environments for open procedures, such as hip arthroplasty.^20,21^ However, technological advancements and increased investment have led to significant improvements in the fidelity of VR environments, making them more applicable to complex, open procedures.^17^

VR training offers several potential advantages over traditional surgical training methods. The gamification potential of VR creates an enjoyable, psychologically safe setting for skill practice, while objective performance outcomes allow trainees to make mistakes safely and improve performance through repeated practice.^22,23^ VR platforms can be affordable and convenient while providing standardised environments for clinical experiential learning with on-demand access.^22^ Additionally, immersive systems allow for the creation of customized simulation curricula tailored to specific requirements, while generating significant amounts of performance data.^22,24^ This data is invaluable for competency-based training, ensuring trainees meet the required standards before operating on patients, identifying those who may need additional training, and encouraging learner engagement.

Studies examining surgical skills in complex open procedures have provided some support for the effectiveness of VR training. For example, Logishetty, Rudran, and Cobb^24^ found that a 6-week VR training programme resulted in superior cadaveric total hip arthroplasty (THA) performance compared to those with only conventional preparatory materials. However, the virtual environment in this study utilised hand-held, motion-tracked controllers that lacked realistic dexterous actions or haptic feedback. Haptic feedback plays a crucial role in surgery, as it delivers essential tactile sensations and proprioceptive information that enables surgeons to achieve precise motor control and develop proficient tissue handling skills.^25-27^ Surgeons have identified this absence of haptic feedback as a major limitation in VR training.^28,29^ Therefore, by integrating haptic realism with the established benefits of gamification, psychological safety, and objective performance data, such systems may enhance the effectiveness of competency-based training and skill transfer. Consequently, this research aimed to explore the potential of haptic-enabled VR technology in accelerating the acquisition of hip arthroplasty skills. It is important, however, to note that VR itself does not directly impart surgical skill acquisition but rather provides an immersive platform facilitating experiential learning through realistic practice environments.

The purpose of this study was to assess whether there was any evidence that haptic-enabled VR technology provided superior hip arthroplasty skill acquisition compared to traditional mentor teaching. To evaluate this, undergraduate medical students’ performance in a SawBone-simulated hemiarthroplasty procedure was assessed. Hip arthroplasty was selected due to its steep learning curve and the growing complexity of skills required for modern techniques, including muscle-sparing and minimally invasive approaches.^30-32^ It was predicted that VR training would lead to improved SawBone performance, as demonstrated by superior technical skills, reduced procedural errors, and faster operative times in VR-trained individuals, reflecting accelerated acquisition of hip arthroplasty skills through immersive practice opportunities.

## Methods

### Participants and Recruitment

Twenty 1st or 2nd year undergraduate medical students (Age: 20 ± 2 years) from the University of Exeter were recruited via an email-distributed recruitment poster. Participants were randomly assigned into two equal-sized groups using stratified block randomization based on their year of study. Students with prior experience observing or assisting in hip surgery were excluded. All participants provided informed consent, and all procedures were approved by the local research ethics committee. Upon completing the study, participants received a certificate signed by an Orthopaedic Consultant (AK) acknowledging their participation.

### Study Design

This was a randomized, controlled, parallel group study of VR training vs a control group. A baseline questionnaire^33^ completed by all participants before training highlighted that there we no differences in procedural knowledge between the groups (*P* = .697). The VR training was overseen by a third-year medical sciences student in a university simulation lab, whereas participants in the control group were trained by a fifth-year medical student (JD) at Exeter and Devon hospital. Training was individualized and participants learned their group assignment upon arrival.

Both groups received the Exeter stem surgical technique guide by Stryker and corresponding training materials.^33^ The steps for the SawBone simulation were identified and matched in both sets of training materials. A Ronguer, which was mentioned in the training materials, was not available in the SawBone simulation; participants were informed and instructed to use a box osteotome instead. After the SawBone assessment, participants in the VR group completed an online questionnaire to provide additional insights on their experience and previous gaming/VR exposure.^33^

## VR Training Protocol

Training began with a live demonstration of the VR module, which included an explanation of the procedural steps and tool functions, followed by their first attempt; visual prompts for instrument position and anatomy were encouraged on the first attempt but became optional for any subsequent attempts. Participants had unlimited attempts within the 60-minute session, skipping steps unrelated to the SawBone simulation. Assistance was provided for VR issues, and questions were limited to those reinforcing the information given in the introduction or relating to VR usage.

**Figure 1.**
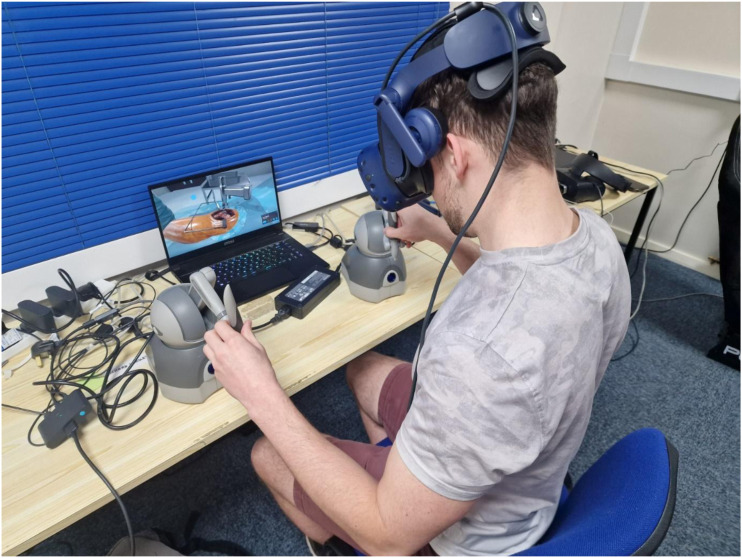
Photo of the VR Setup With the Posterior THA Simulation Module Being Performed

The VR training module used was a posterior THA simulation (Fundamental VR, London, United Kingdom) in training mode (Figure 1). The virtual environment used an HTC VIVE Pro Eye headset (HTC Vive, New Taipei City, Taiwan) and Geomagic Touch haptic feedback controllers (3D Systems, Rock Hill, South Carolina) for interaction with the virtual patient and instruments, allowing users to grip and operate instruments. After training, Participants were instructed not to seek further information until the assessed SawBone simulation was completed.

## Control Training Protocol

Control group participants received a modified hip arthroplasty training PowerPoint, created by the orthopaedic team at the Royal Devon and Exeter Hospital for training purposes.^33^ The trainer briefly reviewed the procedural steps and tool functions while demonstrating instrument use with pre-recorded hip arthroplasty surgery. Participants had 60 minutes to review resources independently, and the trainer addressed questions that reinforced the initial overview. Like the VR group, participants were instructed not to seek additional information after completion of this training.

### SawBone Simulation

**Figure 2.**
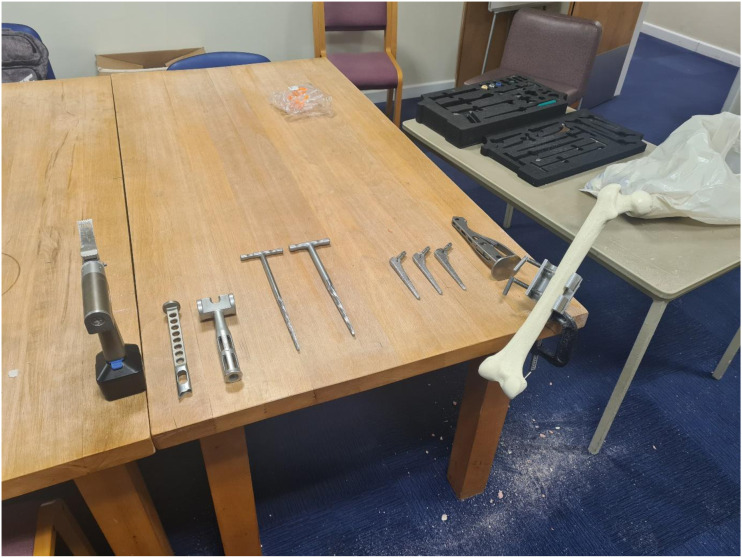
Image of the Equipment Set up for the SawBone Hemiarthroplasty Procedure

After training, participants’ performance was assessed in a SawBone simulated hemiarthroplasty procedure involving femoral head removal, femoral canal opening, and femoral broach insertion. Both training groups were provided with identical equipment setup (Figure 2) and given instructions on the safe and accurate use of the required tools before the task. The Sawbone Simulation was selected as the primary evaluation tool because it provides a standardised and reproducible environment suitable for the objective measurement procedural accuracy, and was readily available to the researchers. Participants were instructed to insert the femoral implant with the top positioned 15 mm below the greater trochanter, while aiming for specific femoral axis (neutral) and anteversion (15° from neutral) targets; explanatory images were provided for clarity.^33^ Each participant had 20 minutes to complete the task. Participants’ performances were recorded with a Galaxy S22 Ultra Smartphone (Samsung, Seoul, South Korea), in an over the shoulder perspective, for blind evaluation by an Orthopaedic Consultant (AK). SawBone performance was evaluated based on participants’ procedural error, technical skill, and operative time.

### Evaluation of Skills

**Figure 3.**
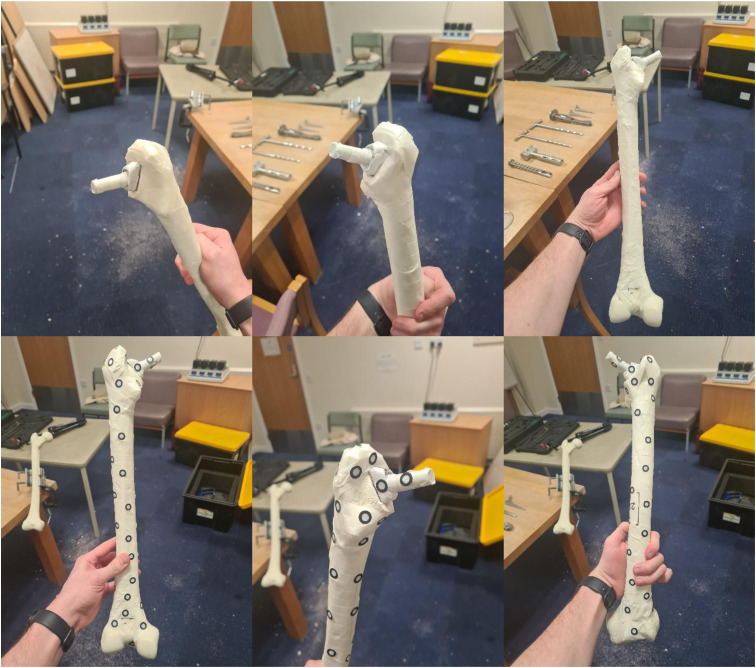
A Series of Images Showing How the Femur was Prepared for 3D Scanning. First, the Femur was Reverse Wrapped in Masking Tape (Top Three Images), Creating a Sticky Surface for the Targeting Stickers to be Placed on and Allowing the Shiny Implant to be Picked up by the 3D Scanner. Location Stickers Were Then Placed at Regular Intervals Around the Bone (Bottom Three Images), which Were Used by the 3D Scanner as Reference Points for Accurate Scanning. A 5 cm Line was Drawn, Enabling the 3D Scanned Image to be Scaled to Its Correct Size in the Blender Software (See Figure 4)

**Figure 4.**
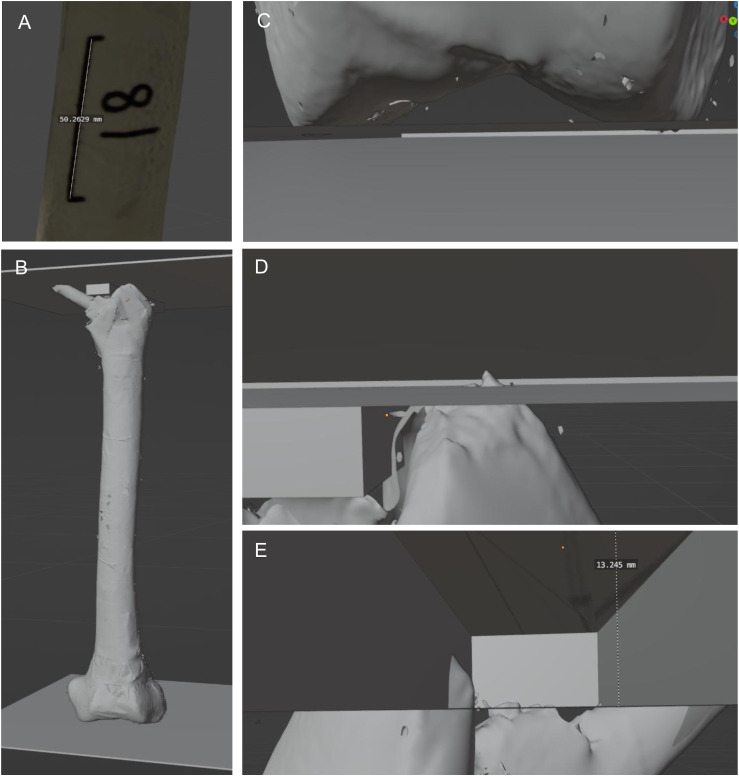
Femoral Implant Depth Measurement. To Measure the Insertion Depth, the Femur was Scaled to the 5 cm Line (A). The Femur was Then Put Into an Upright Position and Three Cubes Were Placed at Particular Landmarks (B). The First Cube was Placed Underneath the Femur, and the Femur was Rotated Until the Lateral and Medial Condyle Touch the Bottom Cube Equally (C). The Bottom of the Second Cube was Placed to Touch the Most Superior Aspect of the Greater Trochanter (D). If the Implant is Superior to the Greater Trochanter, the Bottom of the Second Cube was Placed to Touch the Most Superior Aspect of the Femoral Implant Body. The Third Box was Then Placed Between the Bottom of the Second Box and the Most Superior Aspect of the Femoral Implant Body (or Greater Trochanter if the Implant was Superior); the Diameter of the Cube was Then Measured to Get the Distance Between the Greater Trochanter and the Top of the Implant (E)

Procedural error in the SawBone simulated hemiarthroplasty procedure was assessed using implant depth error (ID_error_) as a surrogate measure for leg length discrepancy; ID_error_ was defined as the distance from the optimal insertion depth, where the top of the femoral implant should be positioned, at a present distance of 15 mm below the tip of the greater trochanter. After completing the procedure, femurs were 3D scanned using a Creaform academia 50 (Creaform, Québec, Canada), method outlined in Figure 3. The resulting images were then imported into Blender (v3.4.1; Blender Foundation, 2022), where they were scaled to their correct size, and insertion depth was measured (method outlined in Figure 4), and ID_error_ calculated.

Operative time was evaluated by categorizing participants into two groups based on ID_error_: successful (ID_error_ ≤ 15 mm), or unsuccessful (ID_error_ > 15 mm) procedure completion. This categorisation was chosen as a leg length discrepancy exceeding 15 mm is typically deemed unacceptable due to its negative impact on patient satisfaction.^34^ The operative time of each group was analysed independently.

An orthopaedic consultant (AK) assessed participants’ technical skills using the provided Global Assessment 5-Point Rating Scale^33^ (adapted from Blumstein et al,^14^ originally developed by Martin et al^35^). The consultant evaluated the videos in a randomised order without any identifiable information, blinded to group condition.

### Statistical Analysis

Statistical analysis was performed using IBM SPSS Statistics (v28.0.1.1; IBM Corp., 2021). Data normality and homogeneity of variance were assessed with the Shapiro-Wilk and Levene’s tests, respectively. Parametric data comparisons were conducted using independent sample t-tests, non-parametric and ordinal data using Mann-Whitney U tests, and categorical data using Pearson chi-squared tests. A two-tailed *P*-value of 0.05 or less indicated a significant difference between the groups. For operative time, Bonferroni correction was applied, resulting in an adjusted alpha level of 0.025. Cohen’s *d* effect sizes were calculated for t-tests. For Mann-Whitney U tests, effect sizes were calculated using the equation: *r* = z-score/√N, where N represents the number of observations.^36^

## Results

### Insertion Depth Error

**Figure 5.**
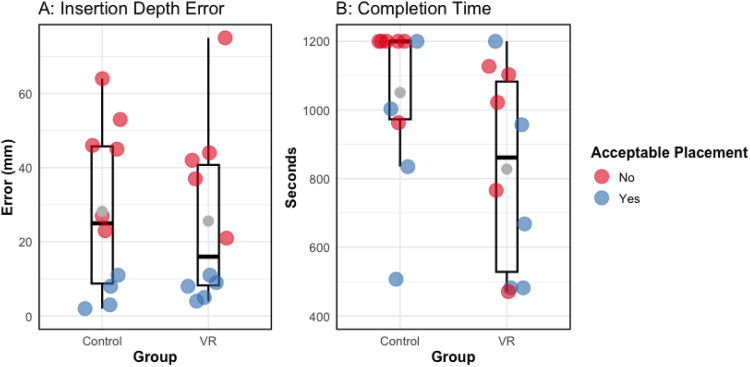
Scatter Plot Showing Participants Individual Implant Insertion Depth Errors (A) and Individual Completion Times (B). The Length of Each Box Represents the Interquartile Range (IQR), the Central Black Line is the Median, the Grey Circle Marker Indicates the Mean. Individual Data Points are Overlaid on the Boxplot. Error Bars Show Standard Error of the Mean

A Shapiro-Wilk test showed that ID_error_ significantly departed from normality, W(20) = 0.898, *P* = .037. A subsequent Mann-Whitney U test indicated no significant differences in ID_error_ between the VR (median = 16 mm, IQR = 35) and control groups (median = 25 mm, IQR = 41), *z* = 0.378, *P* = .705, *r* = 0.085 (Figure 5A). The number of successful procedures (ID_error_ ≤ 15 mm) also did not significantly differ between the groups (VR: 5 of 10, control: 4 of 10), *X*^2^ (1, N = 20) = 0.202, *P* = .653.

### Operative Time

The individual data points for operative time are shown in Figure 5B. For participants with a ID_error_ ≤ 15 mm, a Shapiro-Wilk test did not indicate non-normality in operative time, W(9) = 0.884, *P* = .172. A subsequent t-test revealed no significant difference in operative time between the VR (*M* = 758 s, *SD* = 314) and control (*M* = 886 s, *SD* = 294) groups, *t*(7) = 0.626, *P* = .551, *d* = 0.420.

For participants with a ID_error_ > 15 mm, A Shapiro-Wilk test showed that operative time significantly departed from normality, W(11) = 0.747, *P* = .002. A subsequent Mann-Whitney U test indicated that the VR group (median = 1022 s, IQR = 497) stopped the procedure significantly earlier than the control group (median = 1200 s, IQR = 59), z = 2.298, *P* = .022, *r* = 0.693.

### Technical Skill

**Figure 6.**
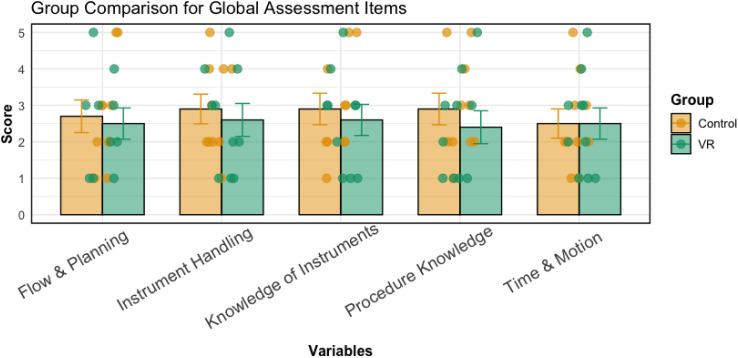
Bar Chart Showing the Mean Scores for all Five Components of the Global Assessment 5-Point Rating Scale. Individual Data Points are Overlaid on the bar Chart. Error Bars Show Standard Error of the Mean

As measured by the Global Assessment 5-Point rating scale evaluated by a blinded orthopaedic consultant, no significant difference in technical skills was found between the VR (median = 13, IQR = 12) and control groups (median = 12, IQR = 10), z = 0.380, *P* = .704, *r* = 0.085 (Figure 6). As anticipated for a scale designed to assess technical skills, individuals who successfully performed the procedure (median = 16, IQR = 13) exhibited notably higher technical skills than their counterparts (median = 10, IQR = 9), z = 2.179, *P* = .029, *r* = 0.487, as determined through this blinded assessment.

## Discussion

This study was the first to assess whether haptic-enabled VR technology could enhance hip arthroplasty skill acquisition in medical students performing a SawBone simulated hemiarthroplasty procedure. While both groups showed a similar level of technical performance as evaluated by a blinded orthopaedic consultant, suggesting that VR may offer a compelling addition to conventional early-stage surgical education, this study highlights some potential limitations of VR training.

Contrary to our prediction, a similar level of ID_error_, technical skill, and successful insertion operative time was observed between real-world and VR-trained groups, indicating that the VR training did not appear to lead to better outcomes in the physical environment than conventional training methods. The technical skills assessments, conducted through blinded evaluation, provide robust evidence for these findings. This result is inconsistent with studies which reported improved hip arthroplasty performance with VR training.^20,21,24^ A possible explanation for the conflicting findings could be attributed to the differences in the training protocols used. For instance, Logishetty, Rudran, and Cobb^24^ implemented a 6-week VR training programme consisting of four 45-60 minute sessions, with additional sessions provided to ensure participants met the criterion levels of VR performance. In contrast, the current study utilized only one hour of simulator training, which may not have been sufficient to observe improvements in hip arthroplasty performance large enough to overtake those in our traditional training group. Alternatively, the discrepancy in results could be due to the unequal levels of training provided to the groups in the Logishetty, Rudran, and Cobb^24^ study, whose VR group received training until a level of proficiency equivalent to expert surgeons’ psychomotor skills was met, whereas the control group received minimal reminders to use preparatory materials independently, without any form of assistance, which does not reflect a realistic surgical training environment.^37^ This potential confound is particularly salient considering that the participants were trainee surgeons with limited available time.^38^ By contrast, our study provided both groups with roughly equal time and engagement from the research team providing the training, strengthening the validity of our comparison and addressing a common confound in prior VR studies where VR groups often received longer or more intensive training than controls. Therefore, further research with well-matched control groups is necessary to determine the efficacy of VR in a range of surgical tasks.

Another potential explanation for conflicting results between the current work and the prior literature may be differences in the virtual environment used. Hooper et al^20^ and Kenanidis et al^21^ used ORamaVR software, which employed hand controllers without haptic feedback. While haptic feedback has proven beneficial for high-precision laparoscopic skills,^26,39,40^ it may not enhance learning curves for more complex tasks.^41^ Few studies have investigated haptic feedback beyond laparoscopic surgery, but a recent study by Xin et al^23^ noted improved cadaveric pedicle screw placement accuracy, success rate, and efficiency with haptic VR compared to traditional teaching methods. However, this procedure involved basic drilling tasks known to benefit from haptic feedback.^25^ In the present study, although one participant found the haptic devices helpful, participants commented on frustrations around glitches, unresponsiveness, and malfunctions.^33^ Furthermore, the haptic arms as fixed input devices posed significant challenges for multiple participants, who encountered difficulties and restrictions with tool positioning and orientation. These technical limitations represent a critical barrier to effective learning, as the rigid positioning constraints prevented natural ergonomic movements. Therefore, it is essential to consider the potential negative implications of fixed input devices in VR training, which do not accurately replicate the tools and dynamics of real-world surgery.^42^ Future work should prioritise the development of more flexible, ergonomically adaptable input devices that better replicate real-world surgical tools and allow for a wider range of natural hand positions and movements. Additionally, addressing technical reliability issues such as glitches and unresponsiveness is paramount before these systems can be effectively integrated into surgical education.

The Cognitive Load Theory (CLT) may explain how the challenges posed by the VR environment in the present study could have negatively impacted learning.^43^ Cognitive load represents the strain a task places on a learner’s cognitive system when they perform it. Difficulties arising from the software or haptic devices result in the allocation of additional cognitive resources to master the technology, thereby increasing cognitive load and hindering performance and any potential VR benefits.^43,44^ Critically, the limited familiarisation time provided in this study (one hour) may have been insufficient for participants to overcome the initial cognitive burden of learning to use the haptic devices effectively. Moreover, poorly calibrated or inaccurate haptic feedback may reinforce incorrect motor patterns or generate unrealistic forces and friction^42,45^ which may be more detrimental than the absence of haptic feedback altogether. Future studies should incorporate extended familiarisation periods with the VR environment and haptic devices before procedural skill assessment, allowing participants to develop technological fluency and reduce cognitive load associated with device operation. This preliminary training phase could include dedicated time for participants to become comfortable with the haptic interface and virtual environment navigation before focusing on surgical skill development.

Our results suggest that VR is an effective tool for acquiring surgical skills through experiential learning, while prior research highlights its potential to be engaging and cost-effective.^22^ Beyond engagement, VR-based training environments offer unique educational advantages. The immersive and interactive nature of VR allows for the development of tailored simulation curricula and provides trainees with unlimited opportunities for deliberate practice in a psychologically safe environment.^22,23^ The addition of haptic feedback can further enhance these benefits by delivering tactile realism and proprioceptive information, supporting the development of precise motor control and proficient tissue-handling skills.^25-27^ However, realising these benefits requires addressing current technical limitations and ensuring adequate familiarisation with the technology. This method is supported by Kopta’s theory,^46^ which describes the acquisition of motor skills and suggests that with frequent hands-on practice and feedback, learners develop an understanding of the task and appropriate motor behaviours, ultimately leading to more seamless and consistent performance patterns (see also Wanzel, Ward and Reznick^47^). Crucially, immersive VR systems, particularly those with haptic feedback, can generate richer and more accurate objective performance data, which can be used to guide competency-based progression, identify trainees in need of further support, and ensure minimum standards are met before progressing to patient care, all while facilitating safe, hands-on experiential learning where skill acquisition occurs through realistic practice opportunities.^22,24^

In our specific procedure, some variability observed among the participants in both groups may be attributed to the absence of a Ronguer during the SawBone procedure. All participants who encountered difficulties in advancing the implant down the femur failed to complete a critical step involving the resection of the lateral-most cortical bone of the neck; a step traditionally performed using a Ronguer in both training resources. While participants had been taught to use the box osteotome for an earlier step in the procedure, they were required to adapt its use to achieve this additional resection. This need for instrument adaptation may have introduced an extra challenge, as procedural tasks typically involve “near transfer” of training where acquired skills are applied in similar, but not identical, contexts as described in Thorndike’s theory of identical elements.^48-50^ Although this form of procedural training is generally easy to learn with a high learning transfer rate,^49^ some participants may have struggled to repurpose the instrument effectively, contributing to performance variability and potentially masking differences attributable to the training methods, particularly given the small sample sizes in this study. While these procedural observations were noted during data collection, they are supported by our finding that individuals who successfully performed the procedure exhibited notably high technical skills than their counterparts (median = 16 vs 10, *P* = .029), as determined through blinded assessment by an orthopaedic consultant, ensuring objective measurement of overall performance despite the variability observed.

A significant strength of this study lies in incorporating haptics into the VR environment. To the best of our knowledge, this is the first investigation into the effectiveness of a haptic-enabled VR platform for facilitating experiential learning of surgical skills within the context of a complex open procedure, such as hip arthroplasty.^14,20,21,24^ This pioneering approach provides valuable insights into the integration of haptics within VR surgical training and emphasizes the need to enhance haptic devices to ensure they are user-friendly and intuitive. The input constraints put in place by haptic devices may, however, hinder performance and undermine the overall efficacy of VR training – particularly in surgical procedures with high degrees of freedom – if the fidelity of the haptic inputs is not sufficiently high.

A key limitation of this study is the small sample size (n = 10 per group), which would allow us to detect only a large effect size. Future studies could test larger samples in order to determine whether VR might have smaller effects on performance (eg, 44 participants per group to detect an effect size of r = 0.3).

Another key limitation of this study was the training process employed. Due to time constraints and hospital room availability, the VR and control group training sessions were administered by different individuals, potentially introducing a trainer effect. VR participants observed a single demonstration of the VR surgery performed by the VR trainer to familiarize them with the proper use of the haptic devices. While the trainer in the control group adopted a role similar to that of a surgeon providing a presentation. These modifications in the training approach may have introduced a potential trainer learning effect, wherein the trainer’s increased experience and familiarity with the training content could have influenced the study’s outcomes. Additionally, trainers might have inadvertently introduced experimenter bias by unintentionally modifying their teaching style in response to participants’ struggles during data collection. To address this limitation, future research should consider implementing standardised training protocols of longer durations that provide consistent and extensive training experiences across all participants. Extended training periods would allow participants to move beyond the initial learning curve associated with technology familiarisation and progress to meaningful skill development, facilitating the transition from conscious, effortful control of the haptic devices to more automatic, fluid movements that better support surgical skill acquisition.^46,47^ This could involve using recorded videos demonstrating the proper utilisation of haptic devices, or conducting a group teaching session specifically focused on their usage. Furthermore, extended training protocols would enable participants to benefit from repeated practice opportunities, which may reveal performance differences that shorter training periods fail to detect, potentially providing clearer evidence of VR’s effectiveness in surgical skill development when technological barriers are adequately addressed through sufficient practice time.^22,23^

## Conclusion

In conclusion, this study represents the first evaluation of haptic-enabled VR technology for surgical training in a complex open procedure such as hip arthroplasty, and highlights the potential use of VR simulators with haptic feedback in surgical training. The results indicate that both traditionally trained and VR-trained groups demonstrated comparable performance in the SawBone hemiarthroplasty procedure, suggesting that VR may serve as a useful adjunct to traditional training, particularly in the early stages of skill acquisition. However, further research comparing haptic vs non-haptic VR is necessary to determine what procedures, and what stage of training, these innovations should be integrated into surgical training programmes. This research should aim to increase the duration and variety of training, both of which are key benefits offered by immersive technologies. The integration of VR technology in surgical education can optimize hospital resources, reduce dependence on cadavers and one-on-one mentoring, and save time and expenses, and these factors are likely to be drivers for the widespread adoption of VR in surgical education.

## Data Availability

All relevant data and code is available online from: https://osf.io/4jpmn/?view_only=8122188df75544cca114606f4b4085cb. All authors have read and approved the final submitted manuscript.
